# Evaluation of [^11^C]CB184 for imaging and quantification of TSPO overexpression in a rat model of herpes encephalitis

**DOI:** 10.1007/s00259-015-3021-x

**Published:** 2015-03-13

**Authors:** David Vállez Garcia, Erik F. J. de Vries, Jun Toyohara, Kiichi Ishiwata, Kentaro Hatano, Rudi A. J. O. Dierckx, Janine Doorduin

**Affiliations:** 1 Department of Nuclear Medicine and Molecular Imaging, University Medical Center Groningen, University of Groningen, PO Box 30.001, 9700 RB Groningen, The Netherlands; 2 Research Team for Neuroimaging, Tokyo Metropolitan Institute of Gerontology, Tokyo, 173-0015 Japan; 3 Department of Clinical and Experimental Neuroimaging, Center for Development of Advanced Medicine for Dementia, National Center for Geriatrics and Gerontology, Obu, Aichi 474-8522 Japan

**Keywords:** PET, TSPO, PBR, Neuroinflammation, Rat

## Abstract

**Purpose:**

Evaluation of translocator protein (TSPO) overexpression is considered an attractive research tool for monitoring neuroinflammation in several neurological and psychiatric disorders. [^11^C]PK11195 PET imaging has been widely used for this purpose. However, it has a low sensitivity and a poor signal-to-noise ratio. For these reasons, [^11^C]CB184 was evaluated as a potentially more sensitive PET tracer.

**Methods:**

A model of herpes simplex encephalitis (HSE) was induced in male Wistar rats. On day 6 or 7 after virus inoculation, [^11^C]CB184 PET scans were acquired followed by ex vivo evaluation of biodistribution. In addition, [^11^C]CB184 and [^11^C]PK11195 PET scans with arterial blood sampling were acquired to generate input for pharmacokinetic modelling. Differences between the saline-treated control group and the virus-treated HSE group were explored using volumes of interest and voxel-based analysis.

**Results:**

The biodistribution study showed significantly higher [^11^C]CB184 uptake in the amygdala, olfactory bulb, medulla, pons and striatum (*p* < 0.05) in HSE rats than in control rats, and the voxel-based analysis showed higher bilateral uptake in the pons and medulla (*p* < 0.05, corrected at the cluster level). A high correlation was found between tracer uptake in the biodistribution study and on the PET scans (*p* < 0.001, *r* ^2^ = 0.71). Pretreatment with 5 mg/kg of unlabelled PK11195 effectively reduced (*p* < 0.001) [^11^C]CB184 uptake in the whole brain. Both, [^11^C]CB184 and [^11^C]PK11195, showed similar amounts of metabolites in plasma, and the binding potential (BP_ND_) was not significantly different between the HSE rats and the control rats. In HSE rats BP_ND_ for [^11^C]CB184 was significantly higher (*p* < 0.05) in the amygdala, hypothalamus, medulla, pons and septum than in control rats, whereas higher uptake of [^11^C]PK11195 was only detected in the medulla.

**Conclusion:**

[^11^C]CB184 showed nonspecific binding to healthy tissue comparable to that observed for [^11^C]PK11195, but it displayed significantly higher specific binding in those brain regions affected by the HSE. Our results suggest that [^11^C]CB184 PET is a good alternative for imaging of neuroinflammatory processes.

## Introduction

Microglia, part of the innate immune system of the central nervous system (CNS), constantly scan the brain for intruding pathogens and contact synapses for neuronal damage. Activation of microglia in response to alterations in the brain microenvironment is a dynamic process [1], characterized by a change in the microglial shape and phagocytic behaviour. All pathological events in the CNS are accompanied by activation of microglia, which acquire distinct functional and phenotypic states during progression of a specific pathology. This responsiveness to brain insults suggests that the microglia have the potential to be used as diagnostic markers of disease state and progression in pathologies such as Alzheimer’s and Parkinson’s diseases, multiple sclerosis and herpes simplex encephalitis (HSE), as well as in stroke, traumatic brain injury and other neuropsychiatric diseases [2–5].

The translocator protein (18 kDa; TSPO), formerly known as the peripheral benzodiazepine receptor, is a transmembrane multimeric protein complex primarily located in the outer mitochondrial membrane of cells [6]. TSPO has been shown to be involved in a variety of cellular functions, including cholesterol transport, steroid hormone synthesis, mitochondrial respiration, mitochondrial permeability transition pore opening, apoptosis and cell proliferation [6–9]. Under normal physiological conditions, overall TSPO expression in the CNS is low and is mainly located in glial cells (astrocytes and microglia), with very low levels in neurons. In pathological processes, TSPO expression is upregulated in glial cells and infiltrating macrophages [10]. Therefore, TSPO has been considered a sensitive marker for the detection of neuroinflammation.

Changes in TSPO expression can be visualized and quantified in vivo using PET. (*R*)-[^11^C]PK11195 has been widely used as a PET probe for imaging TSPO expression in animal models and humans with various CNS diseases, including glioma, stroke, HSE and neurodegenerative disorders such as Alzheimer’s disease, multiple sclerosis, amyotrophic lateral sclerosis and Parkinson’s disease [5, 8, 11, 12]. However, (*R*)-[^11^C]PK11195 suffers from several limitations, including poor signal-to-noise ratio (mainly due to its low binding potential to TSPO and high levels of nonspecific binding), highly variable kinetic behaviour and apparent lack of sensitivity in detecting low levels of microglial activation [12, 13].

Because of these limitations of (*R*)-[^11^C]PK11195, there has been an effort to develop more sensitive and selective PET ligands for imaging activated microglia. Several chemically diverse ligands with high affinity for TSPO have been found (detailed information is available elsewhere [5, 14, 15]). These ligands include imidazopyridine acetamide derivatives (e.g. [^11^C]CLINME [16]), indole acetamides (e.g. [^11^C]SSR180575 [17]), pyrazolopyrimidines (e.g. [^11^C]DPA-713 and [^18^F]DPA-714 [18]) and phenoxy arylamides (e.g. [^11^C]PBR28 [19], [^11^C]DAA1106 [20]). However, most of these new TSPO ligands are still in the early stages of investigation, and in contrast to (*R*)-[^11^C]PK11195, suffer from mixed-affinity binding due to a TSPO polymorphism in humans [21, 22], which seriously complicates their use in clinical studies.

In the search for a better alternative to (*R*)-[^11^C]PK11195, the novel imidazopyridine compound [^11^C]CB184 was developed [23]. [ ^11^C]CB184 shows 7.9 times higher TSPO affinity than (*R*)-[^11^C]PK11195 (*K*_i_ = 0.54 nM and 4.27 nM, respectively). Furthermore, [^11^C]CB184 shows lower lipophilicity than (*R*)-[^11^C]PK11195 (log*P* = 2.06 and 2.54, respectively). As a result, [^11^C]CB184 shows higher uptake in TSPO-rich regions in normal mice (cerebellum and olfactory bulb), and comparable inflammation-induced binding in 6-hydroxydopamine-injured striatum in rats, as compared with (*R*)-[^11^C]PK11195. In the present study, [^11^C]CB184 was further evaluated in a rat model of HSE [18]. The study was divided into two parts. First, the characteristics of [^11^C]CB184 were investigated in ex vivo biodistribution and in vivo PET imaging studies in healthy and HSE rats. In the second part, a pharmacokinetic analysis was performed comparing [^11^C]CB184 and (*R*)-[^11^C]PK11195.

## Materials and methods

### Rats

Male outbred Wistar-Unilever rats (*n* = 45) at 6 – 8 weeks of age (weight 282 ± 25 g) were obtained from Harlan (Horst, The Netherlands). After arrival, the rats were allowed to acclimatize for at least 7 days. Rats were individually housed in Makrolon cages on a layer of wood shavings in a room at constant temperature (21 ± 2 °C) and a 12-h light/night regime. Commercial chow and water were available ad libitum. The distribution of the rats across the groups is detailed in Table 1. In summary, rats were divided into eight groups, used in the PET SUV and ex vivo biodistribution studies (control, seven rats; HSE, seven rats; control pretreated with PK11195, five rats; and HSE pretreated with PK11195, five rats), and the pharmacokinetic analysis of [^11^C]CB184 (control, five rats; and HSE, six rats) and (*R*)-[^11^C]PK11195 (control, five rats; and HSE, five rats).

**Table 1:** Experimental groups of rats, injected activities and injected masses (mean ± SD)

		Control groups	HSE groups	Injected activity (MBq)	Injected mass (nmol)
PET SUV and ex vivo biodistribution					
[^11^C]CB184	Scan 30 min	4	4	17.2 ± 14.6	2.35 ± 0.81
	Scan 60 min	3	3	48.9 ± 8.2	1.12 ± 0.29
	Scan 30 min + pretreated with PK11195	3	3	11.3 ± 7.6	3.08 ± 1.09
	Scan 60 min + pretreated with PK11195	2	2	41.2 ± 3.8	1.77 ± 0.73
Pharmacokinetic analysisp					
[^11^C]CB184	Scan 60 min + blood sampling	5	6	68.7 ± 22.7	2.74 ± 1.44
(*R*)-[^11^C]PK11195	Scan 60 min + blood sampling	5	5	85.3 ± 20.8	2.27 ± 0.89

All animal experiments were performed according to the Dutch Law for Animal Welfare, and were approved by the Institutional Animal Care and Use Committee of the University of Groningen (DEC 6264A).

### Tracer synthesis

**Fig. 1:**
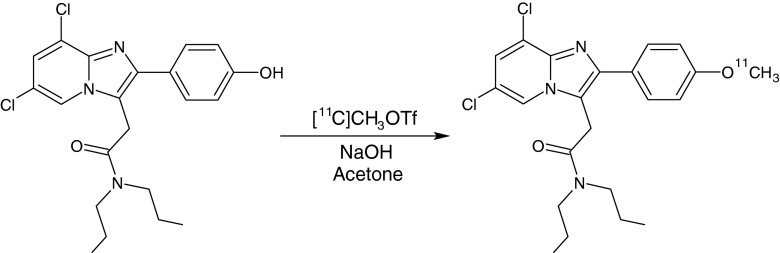
Radiosynthesis of [^11^C]CB184

[^11^C]CB184 was labelled by bubbling [^11^C]methyl triflate with helium gas at 30 ml/min into a solution of 0.25 mg *N*-propyl-2-{6,8-dichloro-2-(4-methoxyphenyl)imidazo[1,2-a]pyridin-3-yl}acetamide (precursor, CB185) and 5 μl 1 M NaOH in 0.25 ml acetone (Fig. 1). When the trapping of [^11^C]methyl triflate was complete, the reaction mixture was diluted with 0.3 ml of water and 1.4 ml of HPLC eluent (55 % aqueous acetonitrile). The reaction mixture was purified by HPLC using a SymmetryShield C18 column (5 μm, 7.8 mm inner diameter, 300 mm length) and acetonitrile/water (55/45) as the eluent (flow 4 ml/min). The radioactive product with a retention time of 12 – 13 min was collected. The product was diluted with 15 ml water and passed through an Oasis HLB 30-mg (1 ml) cartridge. The cartridge was washed with 5 ml water and subsequently eluted with 0.7 ml ethanol and 4.5 ml 0.9 % NaCl. The product was obtained in 42 ± 7 % radiochemical yield (*n* = 14). Quality control was performed by UPLC, using a Waters Acquity H-class UPLC system with a Berthold FlowStar LB 513 radioactivity detector, and a Waters Aquity UPLC C18 BEH phenyl column (1.7 μm, 3.0 × 50 mm). The product was eluted with 40 % acetonitrile in water at a flow rate of 0.8 ml/min. The UV signal was measured at a wavelength of 254 nm. The retention time of the precursor was 2.4 min, and the retention time of [^11^C]CB184 was 5.3 min. The radiochemical purity of [^11^C]CB184 was 99.2 ± 0.9 % and the specific activity 60 ± 25 GBq/μmol. For in vivo imaging, the required dose of the formulated product was dispensed and diluted with saline to a final volume of 6.2 ± 0.6 ml. The concentration of ethanol in the administered product was always <10 %.

The synthesis of (*R*)-[^11^C]PK11195 was as reported in detail elsewhere [18].

### HSV-1 inoculation

HSE rats were prepared as described previously [18]. Briefly, a herpes simplex virus type 1 (HSV-1) strain was obtained from a clinical isolate, cultured in Vero cells, and assayed for plaque-forming units (PFU) per millilitre. The rats were slightly anaesthetized with 5 % isoflurane and inoculated with HSV-1 by administration of 100 μl of phosphate-buffered saline (PBS) with 1 × 10^7^ PFU of virus into the nostrils using a micropipette (50 μl per nostril). Healthy control rats were treated similarly administering 100 μl of PBS without the virus. After inoculation, clinical symptoms were scored daily in all rats by the same observer.

### PET imaging and ex vivo biodistribution

**Fig. 2:**
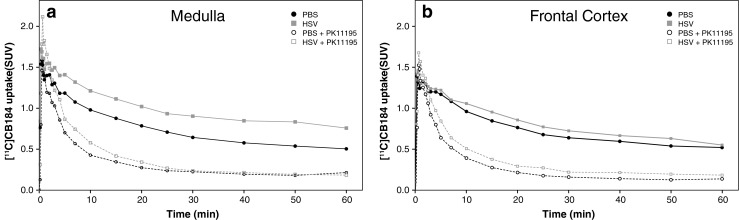
[^11^C]CB184 time–activity curve of two representative regions: medulla (**a**) and frontal cortex (**b**)

PET scans were performed using a microPET Focus 220 camera (Siemens Medical Solutions Inc., Malvern, PA) on either day 6 or day 7 after inoculation with HSV-1 [18], depending on the severity of clinical symptoms. The rats were anaesthetized with 5 % isoflurane mixed with medical air at a flow rate of 2 ml/min. After induction, anaesthesia was maintained with 1.5 – 2 % of isoflurane. The anaesthetized rats were positioned in the camera in a supine position with the head in the field of view. The PET tracer [^11^C]CB184 was manually injected via the penile vein, and at the same time a dynamic 60-min scan was started. Injected tracer doses and injected masses are summarized in Table 1. Visual assessment of tracer uptake in the 60-min scans showed no substantial differences in uptake over time after the first 30 min (Fig. 2). Therefore, it was decided to scan the remaining animals with a 30-min scan. For 30 min the animals were kept under anaesthesia on the operation table after injection of the PET tracer into the penile vein, and were then placed in the scanner for a 30-min dynamic scan. A transmission scan was obtained in all rats using a ^57^Co point source for attenuation and scatter correction. In the pretreated group, 5 mg/kg unlabelled PK11195 (Sigma-Aldrich, St. Louis, MO) in dimethyl sulphoxide (DMSO) at a concentration of 10 mg/ml was administered via a tail vein 5 min before injection of the PET tracer.

After the scans (approximately 75 min after tracer injection), the rats were killed by extirpation of the heart while under deep anaesthesia. The brain and peripheral organs were excised and dissected, and blood was centrifuged to collect a plasma sample. Tissues were weighed and radioactivity was measured in a gamma counter (LKB Wallac, Turku, Finland). Tracer uptake in each region was corrected for the injected tracer dose and body weight and expressed as standardized uptake value (SUV), which was defined as: radioactivity (MBq/cm^3^)/[injected dose (MBq)/body weight (g)].

### PET imaging with arterial blood sampling and blood processing

PET scans with arterial blood sampling were performed in a different set of rats to generate input for pharmacokinetic modelling. For the arterial blood sampling a cannula was inserted into the femoral artery after induction of anaesthesia. The femoral vein was additionally cannulated for PET tracer injection. After cannulation, the rats were positioned in the camera and a transmission scan was acquired using a ^57^Co point source. The PET tracer ([^11^C]CB184 or (*R*)-[^11^C]PK11195) was injected over 1 min using an automatic pump at a speed of 1 ml/min, and a 60-min dynamic PET scan was started. A 0.1 ml blood sample of was taken at 0, 5, 10, 15, 20, 30, 45, 60, 75, 90, 120, 180, 300, 450, 600, 900, 1,800 and 3,600 s after injection. A larger blood sample of 0.5 ml was taken at three time points (5, 15, 30 and/or 60 min) for metabolite analysis. After collection of each blood sample, heparinized saline was injected to prevent large changes in blood pressure. A 25 μl aliquot of whole blood was extracted from each sample for radioactivity measurement. The remainder of each sample was centrifuged at 13,000 rpm (15,996 *g*) for 8 min, and 25 μl plasma was taken for radioactivity measurement. The radioactivity in blood and plasma was measured with a gamma counter.

### PET image reconstruction and analysis

The list-mode data from the emission scan were reconstructed into three frames of 10 min for the dynamic 30-min scans, and into 21 frames (6 × 10 s, 4 × 30 s, 2 × 60 s, 1 × 120 s, 1 × 180 s, 4 × 300 s, and 3 × 600 s) for the 60-min dynamic scans. Emission sinograms were iteratively reconstructed (OSEM 2D, four iterations, and 16 subsets) after being normalized and corrected for attenuation and decay of radioactivity. PET images were analysed using VINCI 4.22 software (Max Planck Institute for Neurological Research, Cologne, Germany). Scans were automatically registered to a functional (*R*)-[^11^C]PK11195 template [24], which was spatially aligned with a stereotaxic T2-weigthed MRI template in Paxinos space [25], facilitating the accurate reporting of results and enabling the use of predefined standard-space atlas structures. Volumes of interest (VOI) for the different brain regions were defined by addition of previously constructed regions [24]. Brain radioactivity concentrations were calculated from these regions of interest to generate time–activity curves (TACs). The TACs were corrected for injected dose and animal body weight, and expressed as SUV. Based on visual analysis of the TACs, it was decided to use the last 10-min frame of the scans obtained without arterial blood sampling for further SUV analysis of differences between groups, because at this point tracer uptake reached a plateau (see Fig. 2). No significant differences were observed between the 30-min and the 60-min scans, and their data were combined.

### Metabolite analysis

Radioactive metabolites in plasma were measured using the extra blood samples taken at three time points during each scan (5, 15, 30 and/or 60 min). After centrifugation and collection of the plasma sample for radioactive measurement, as described above, the remaining plasma was diluted with acetonitrile (1.5 times the amount of plasma) and vortex-mixed. The plasma suspension was centrifuged at 5,300 rpm (3,030 *g*) for 3 min, and divided into supernatant and pellet. The supernatant was filtered through a Millipore filter (Millex-HV 4-mm syringe filter, pore size 0.45 μm) and was then analysed by HPLC using an Alltima RP-C18 column (5 μm, 10 × 250 mm) and 60/40 acetonitrile/water at a flow of 4 ml/min for [^11^C]CB184 or 70/30/0.5 acetronitrile/water/triethylamine at a flow of 5 ml/min for (*R*)-[^11^C]PK11195 as the eluent. Fractions of 30 s were collected and measured in the gamma counter.

The percentage of metabolites in plasma was calculated by fitting an exponential function to the values obtained from the HPLC analysis. The plasma values obtained from the blood samples during the PET scan were then corrected for the presence of these metabolites, and used together with the whole-blood curve for further analysis. Metabolite corrected plasma TACs were fitted for each individual rat using iterative nonlinear least-squares fitting to the biexponential equation: SUV_*t*_ = *A*e^−*αt*^ + *B*e^−*βt*^, where SUV_*t*_ is the plasma SUV at time *t*, *α* and *β* are the apparent distribution and elimination rate constants (s^−1^), respectively, and *A* and *B* are the corresponding zero-time intercepts. The weighting factors were the reciprocals of the plasma SUV squared. Distribution and elimination half-lives (seconds) were calculated as the natural logarithm of 2 divided by *α* and *β*, respectively.

To determine if radioactive metabolites of [^11^C]CB184 entered the brain, the brain of one control rat and one HSE rat were isolated 30 min after injection and homogenized with a solution of ice-cold acetonitrile (3 ml). The homogenate was centrifuged at 6,000 rpm (3,461 *g*) for 10 min. The supernatant was then collected and processed by HPLC, as described above.

### Pharmacokinetic analysis

The TACs of those rats in which blood sampling was performed, together with their corresponding whole-blood and metabolite-corrected plasma curves, were used for pharmacokinetic modelling using PMOD v3.3 (PMOD Technologies, Zürich, Switzerland). A preliminary Logan graphical analysis [26] and a Patlak graphical analysis [27] of tracer kinetics were performed to determine if the [^11^C]CB184 showed reversible or irreversible behaviour. Visual inspection showed a better fit for Logan graphical analysis. Therefore, quantification of [^11^C]CB184 and [^11^C]PK11195 binding was performed with Logan graphical analysis using a delay time of 15 min to calculate the distribution volume (*V*_T_). The reversible two-tissue compartment model (2TCMR) with *K*_1_/*k*_2_ fixed to the whole cortex value [28] and a fixed blood volume of 3 % were used to calculate the *V*_T_ and nondisplaceable binding potential (BP_ND_, calculated as *k*_3_/*k*_4_ [29]).

### Statistical analysis

The results are presented as means ± standard deviation (SD). Statistical analysis was performed using IBM SPSS Statistics 20. Differences between groups were analysed using the independent samples *t*-test, and considered to be significant at *p* < 0.05.

#### Voxel-based analysis

Voxel-based analysis was performed using SPM8 (Wellcome Department of Cognitive Neurology, University College London, UK) and the SAMIT toolbox [24]. A two-sample *t*-test (control rats vs. HSE rats) was performed on [^11^C]CB184 SUV images, obtained from the last 10-min frame of the 30-min and 60-min PET scans without blood sampling. The analysis was done without global normalization or a threshold. Images were smoothed with a 1.2 mm isotropic Gaussian kernel. For interpretation of group differences, T-map data were interrogated at *p* < 0.005 (uncorrected) and an extent threshold of 200 voxels. Only clusters with *p* < 0.05 corrected for family-wise error were considered significant.

## Results

### [^11^C]CB184 characteristics

#### Ex vivo biodistribution

The ex vivo biodistribution study was performed to determine the uptake of [^11^C]CB184 in four experimental groups: control rats, HSE rats, and control or HSE rats pretreated with unlabelled PK11195. The results of the ex vivo biodistribution study are expressed as mean SUV ± SD. Table 2 shows the ex vivo biodistribution in the brain and peripheral organs of the different groups approximately 75 min after tracer injection. Whole-brain uptake of [^11^C]CB184 was significantly higher in HSE rats than in control rats (0.99 ± 0.21 vs. 0.66 ± 0.16, *p* = 0.006). Uptake of [^11^C]CB184 in HSE rats, as compared with control rats, was significantly higher in the amygdala (0.52 ± 0.21 vs. 0.30 ± 0.08, *p* = 0.04), olfactory bulb (1.52 ± 0.22 vs. 1.06 ± 0.15, *p* < 0.001), medulla (1.51 ± 0.57 vs. 0.59 ± 0.14, *p* = 0.001), pons (1.26 ± 0.52 vs. 0.63 ± 0.08, *p* = 0.008) and striatum (0.45 ± 0.15 vs. 0.28 ± 0.07, *p* = 0.03). [^11^C]CB184 binding to TSPO was blocked by administration of unlabelled PK11195 5 min before tracer injection, resulting in a significantly lower uptake of [^11^C]CB184 in all brain regions of HSE and control rats, except in cingulate/frontopolar and frontal cortices of control rats. When the whole brain was considered, a highly significant difference between blocked and nonblocked groups was found in both control and HSE rats (*p* < 0.001).

**Table 2:** Ex vivo biodistribution of [^11^C]CB184, expressed as SUV (mean ± SD) approximately 75 min after injection in control (PBS-treated) rats, HSE rats (infected with HSV-1), and rats pretreated with 5 mg/kg of PK11195 5 min before [^11^C]CB184 injection

	Control group (*n* = 7)	HSE group (*n* = 7)	PBS + PK11195 group (*n* = 5)	HSE + PK11195 group (*n* = 5)
Brain				
Amygdala/piriform cortex	0.30 ± 0.08	0.52 ± 0.21*	0.10 ± 0.05***	0.08 ± 0.06**
Olfactory bulb	1.06 ± 0.15	1.52 ± 0.22***	0.20 ± 0.08***	0.17 ± 0.11***
Cerebellum	0.52 ± 0.06	0.84 ± 0.41	0.13 ± 0.08***	0.11 ± 0.06**
Cingulate/frontopolar cortex	0.36 ± 0.13	0.46 ± 0.21	0.24 ± 0.17	0.10 ± 0.05**
Entorhinal cortex	0.36 ± 0.13	0.57 ± 0.39	0.09 ± 0.07**	0.10 ± 0.05*
Frontal cortex	0.44 ± 0.44	0.39 ± 0.09	0.10 ± 0.07	0.08 ± 0.05***
Hippocampus	0.39 ± 0.18	0.55 ± 0.21	0.12 ± 0.08*	0.09 ± 0.05***
Medulla	0.59 ± 0.14	1.51 ± 0.57**	0.12 ± 0.07***	0.13 ± 0.06***
Parietal/temporal/occipital cortex	0.32 ± 0.06	0.41 ± 0.14	0.10 ± 0.06***	0.10 ± 0.05***
Pons	0.63 ± 0.08	1.26 ± 0.52**	0.12 ± 0.08***	0.12 ± 0.10***
Striatum	0.28 ± 0.07	0.45 ± 0.15*	0.11 ± 0.07**	0.09 ± 0.06***
Whole brain	0.66 ± 0.16	0.99 ± 0.21**	0.15 ± 0.08***	0.08 ± 0.08***
Peripheral organs				
Adrenals	14.84 ± 5.45	13.86 ± 4.42	7.72 ± 5.20*	8.14 ± 7.09
Bone	1.00 ± 0.26	0.98 ± 0.14	0.31 ± 0.19***	0.39 ± 0.24***
Colon	3.38 ± 0.97	3.29 ± 0.81	0.42 ± 0.24***	0.49 ± 0.36***
Fat	0.43 ± 0.21	0.57 ± 0.39	0.48 ± 0.18	0.72 ± 0.49
Heart	22.81 ± 2.80	23.69 ± 2.71	0.68 ± 0.40***	0.77 ± 0.53***
Ileum	6.41 ± 3.07	6.20 ± 2.67	2.31 ± 1.15*	2.74 ± 1.77*
Kidney	12.52 ± 2.22	13.17 ± 2.25	0.72 ± 0.44***	1.12 ± 0.75***
Liver	7.57 ± 1.64	7.97 ± 1.73	11.28 ± 5.43	10.71 ± 9.25
Lung	18.39 ± 2.20	22.53 ± 2.41**	0.95 ± 0.57***	1.62 ± 0.98***
Pancreas	3.55 ± 0.52	4.23 ± 0.76	0.57 ± 0.34***	0.62 ± 0.37***
Plasma	0.18 ± 0.08	0.28 ± 0.24	0.27 ± 0.15	0.19 ± 0.04
Red blood cells	0.19 ± 0.18	0.13 ± 0.01	0.11 ± 0.05	0.11 ± 0.07
Spleen	13.23 ± 2.64	11.76 ± 1.42	0.70 ± 0.47***	0.71 ± 0.55***
Stomach	5.04 ± 0.82	5.88 ± 1.87	0.70 ± 0.46***	0.82 ± 0.60***
Submandibularis	4.28 ± 1.02	4.89 ± 0.75	0.70 ± 0.46***	0.79 ± 0.56***
Testis	0.98 ± 0.11	0.88 ± 0.12	0.30 ± 0.24***	0.29 ± 0.21***
Thymus	3.36 ± 0.58	3.23 ± 0.33	0.72 ± 0.44***	0.74 ± 0.46***
Trachea	5.56 ± 1.50	6.38 ± 3.30	0.81 ± 0.35***	1.02 ± 0.70**

*p < 0.05, **p < 0.01, ***p < 0.001, HSE vs. control groups, and groups pretreated with PK11195 vs. the same group without pretreatment

The ex vivo biodistribution study in peripheral organs showed a high uptake of [^11^C]CB184 in TSPO-expressing organs, including the adrenal glands, heart, kidney, lungs and spleen. [^11^C]CB184 uptake was significantly higher in the lungs of HSE rats than in control rats (22.53 ± 2.41 vs. 18.39 ± 2.20, *p* = 0.006). [^11^C]CB184 uptake was effectively blocked by pretreatment with unlabelled PK11195, resulting in a significant reduction of uptake in almost all the tissues except the adrenals in the control group, and in fat, plasma and red blood cells in both HSE and control rats. The mean [^11^C]CB184 uptake in the liver was higher after administration of unlabelled PK11195, but this increase did not reach statistical significance.

#### PET imaging: VOI-based analysis

In order to assess the ability of [^11^C]CB184 to visualize the TSPO overexpression, healthy control and HSE rats were imaged using PET. The SUVs obtained from the last 10 min of the 30-min and 60-min PET scans (without blood sampling) are shown in Table 3. Uptake of [^11^C]CB184 in whole brain was significantly higher in HSE rats than in control rats (0.52 ± 0.08 vs. 0.41 ± 0.08, *p* = 0.02). For the predefined brain regions (VOI), uptake was significantly higher in the medulla (0.86 ± 0.27 vs. 0.47 ± 0.08, *p* = 0.003), pons (0.73 ± 0.17 vs. 0.44 ± 0.11, *p* = 0.002) and striatum (0.38 ± 0.06 vs. 0.29 ± 0.09, *p* = 0.04). [^11^C]CB184 uptake in rats pretreated with unlabelled PK11195 was significantly lower in all brain regions (*p* < 0.01).

**Table 3:** [^11^C]CB184 uptake, expressed as SUV (mean ± SD), obtained from the PET scan acquired for 50 – 60 min after [^11^C]CB184 injection in control (PBS-treated) rats, HSE rats (infected with HSV-1), and rats pretreated with 5 mg/kg of PK11195 5 min before [^11^C]CB184 injection

	Control group (*n* = 7)	HSE group (*n* = 7)	PBS + PK11195 group (*n* = 5)	HSE + PK11195 group (*n* = 5)
Amygdala	0.43 ± 0.11	0.49 ± 0.14	0.16 ± 0.02***	0.16 ± 0.05***
Olfactory bulb	0.84 ± 0.13	0.96 ± 0.18	0.21 ± 0.03***	0.26 ± 0.03***
Cerebellum	0.48 ± 0.08	0.62 ± 0.17	0.14 ± 0.02***	0.14 ± 0.03***
Frontal cortex	0.44 ± 0.12	0.42 ± 0.13	0.14 ± 0.02***	0.18 ± 0.03**
Remaining cortices	0.40 ± 0.07	0.45 ± 0.06	0.14 ± 0.01***	0.15 ± 0.02***
Hippocampus	0.31 ± 0.09	0.40 ± 0.11	0.13 ± 0.03**	0.12 ± 0.03***
Hypothalamus	0.47 ± 0.15	0.56 ± 0.10	0.16 ± 0.03***	0.18 ± 0.05***
Medulla	0.47 ± 0.08	0.86 ± 0.27**	0.19 ± 0.04***	0.19 ± 0.06***
Midbrain	0.33 ± 0.09	0.42 ± 0.12	0.11 ± 0.02***	0.13 ± 0.03***
Pons	0.44 ± 0.11	0.73 ± 0.17**	0.18 ± 0.03***	0.19 ± 0.05***
Septum	0.29 ± 0.12	0.40 ± 0.16	0.10 ± 0.01**	0.12 ± 0.03**
Striatum	0.29 ± 0.09	0.38 ± 0.06*	0.11 ± 0.03**	0.17 ± 0.05***
Thalamus	0.26 ± 0.07	0.33 ± 0.07	0.12 ± 0.02**	0.11 ± 0.03***
Whole brain	0.41 ± 0.08	0.52 ± 0.08*	0.14 ± 0.01***	0.15 ± 0.02***

*p < 0.05, **p < 0.01, ***p < 0.001, HSE vs. control groups, and groups pretreated with PK11195 vs. the same group without pretreatment

**Fig. 3:**
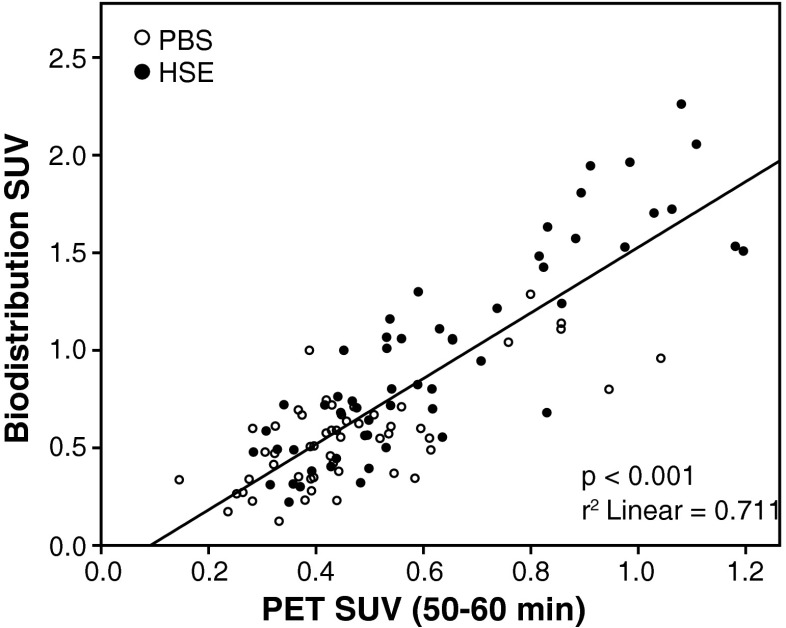
Correlation between [^11^C]CB184 SUV values determined ex vivo and those obtained from the PET scan, from control and HSE rats

TACs of two representative brain regions are shown in Fig. 2. Pretreatment with unlabelled PK11195 reduced the uptake of [^11^C]CB184 in the same manner in control rats and in HSE rats. There was a good correlation between the biodistribution values and the SUVs from PET scans acquired 50 – 60 min after [^11^C]CB184 injection (*p* < 0.001, *r* ^2^ = 0.71; Fig. 3).

#### PET imaging: voxel-based analysis

**Fig. 4:**
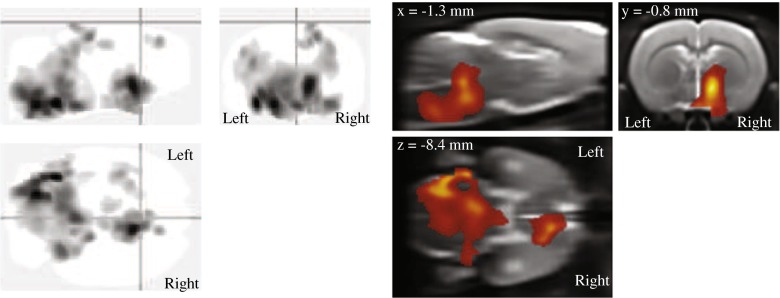
[^11^C]CB184 voxel-based analysis. Uptake of [^11^C]CB184 is significantly higher in HSE rats than in control rats (cluster-level *p* < 0.05, corrected for family-wise error). These regions correspond to the pons and medulla bilaterally, and the thalamus, hypothalamus, internal capsule, substantia innominata, globus pallidus, bed nucleus of the stria terminalis, and diagonal band of the right side. *Left* Standard ‘glass brain’ output in SPM. *Right* Overlay of the results on the MRI template

**Fig. 5:**
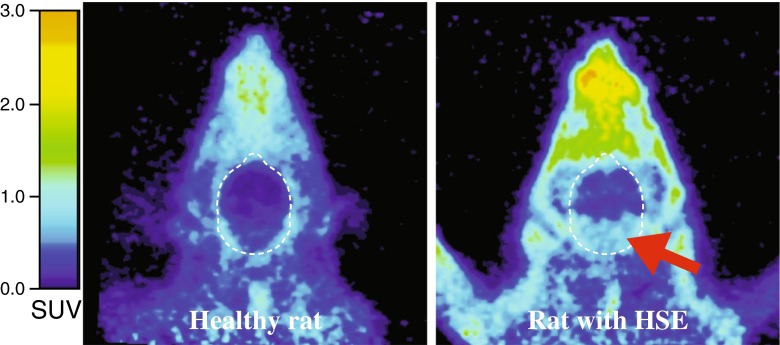
Transaxial [^11^C]CB184 PET images of the head of a healthy control rat and a rat with herpes simplex encephalitis (HSE) (*arrow* increased uptake of [^11^C]CB184 in the region of the pons and medulla)

The results of the voxel-based analysis are shown in Fig. 4 and Table 4. Uptake of [^11^C]CB184 in several brain regions was significantly higher in HSE rats than in control rats (cluster-level *p* < 0.05, corrected for family-wise error). This higher uptake was bilateral for the pons and medulla (Fig. 5), with the maximum difference in uptake located in the left ventral cochlear nucleus (Paxinos coordinates *x*,*y*,*z* = −4.5,−9.4,−9, and *x*,*y*,*z* = −4,−10.6,−9.2) and the left reticular formation (*x*,*y*,*z* = −2.5,−11.6,−9.2). Also, an asymmetrically higher uptake was found in the right hemisphere only in the thalamus, hypothalamus, internal capsule, substantia innominata, globus pallidus and diagonal band, with the maximum difference in uptake located in the right bed nucleus of the stria terminalis (*x*,*y*,*z* = 1.3,−0.8,−7.2, and *x*,*y*,*z* = 0.9,−1.2,−9.2) and the right lateral hypothalamic area (*x*,*y*,*z* = 2.1,−1.2,−8.2).

**Table 4:** [^11^C]CB184 voxel-based analysis (statistically significant results)

Peak probability level		Cluster level			Paxinos coordinates (mm)		
*p* value	Threshold	Family-wise error correction	Uncorrected	Voxels	*x*	*y*	*z*
0.005	200 voxels	<0.001	<0.001	26,598	−4.5	−9.4	−9
					−2.5	−11.6	−9.2
					−4	−10.6	−9.2
		0.005	0.001	4,357	1.3	−0.8	−7.2
					2.1	−1.2	−8.2
					0.9	−1.2	−9.2

### Comparison of [^11^C]CB184 and (*R*)-[^11^C]PK11195

#### Plasma clearance

**Fig. 6:**
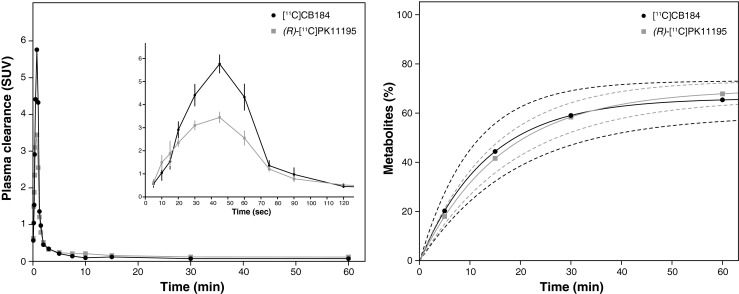
*Left* Plasma clearance corrected for metabolites (SUV ± standard error) of [^11^C]CB184 and (*R*)-[^11^C]PK11195 during the 60 min dynamic PET scan (*inset* expanded view of the first 120 s). *Right* Curves showing the percentages of metabolites present in plasma from the time of injection until the end of the PET scan (*dashed lines* 95 % confidence intervals)

Figure 6 shows the mean TACs of plasma corrected for the percentage of metabolites for [^11^C]CB184 and (*R*)-[^11^C]PK11195 following intravenous injection. A significant difference was found at the peak tracer concentration in plasma during the distribution phase (*p* < 0.003 at 45 s). No significant difference was found during the elimination phase. The distribution half-lives were 17 ± 7 s and 22 ± 7 s, and the elimination half-lives were 44 ± 26 min and 65 ± 46 min for [^11^C]CB184 and (*R*)-[^11^C]PK11195, respectively.

The metabolite concentrations in plasma indicated a similar metabolic rate for both tracers, with 50 % of parent tracer still present in plasma at about 21 min after tracer injection. The amount of [^11^C]CB184 metabolites found in the brain (including the blood component of the brain) at 30 min after tracer injection was 1.2 % in control rats and 1.3 % in HSE rats, suggesting that metabolites do not cross the blood–brain barrier (BBB).

#### Kinetic modelling

**Fig. 7:**
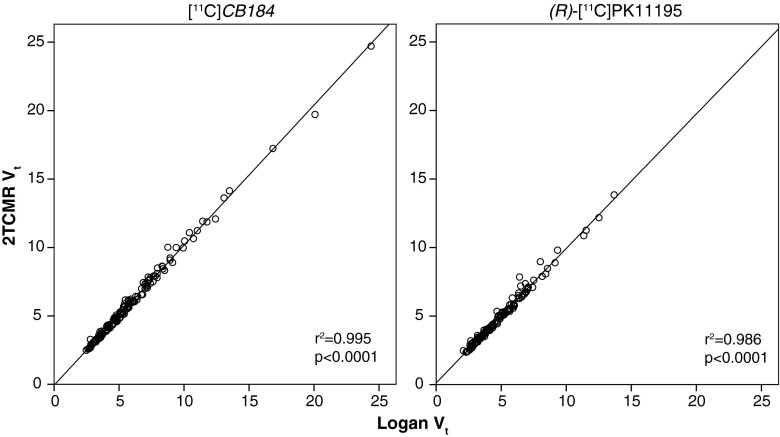
Correlation between distribution volume (*V*_T_) determined by the reversible two-tissue compartment model (2TCMR) and *V*_T_ for [^11^C]CB184 and (*R*)-[^11^C]PK11195 determined by Logan graphical analysis

**Fig. 8:**
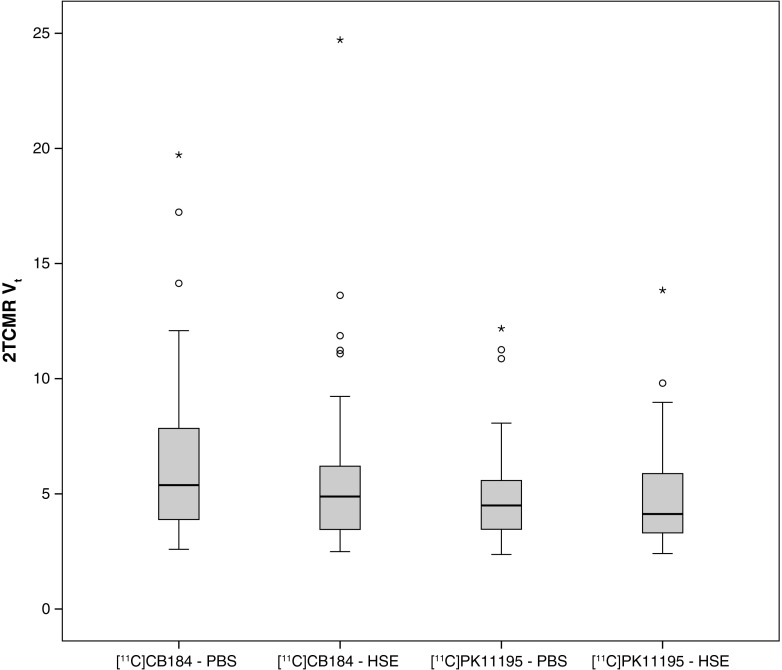
Distribution volume (*V*_T_) values for [^11^C]CB184 and (*R*)-[^11^C]PK11195 in control rats (*PBS*) and rats infected with HSV-1 (*HSE*) determined using the reversible two-tissue compartment model (2TCMR)

For both [^11^C]CB184 and (*R*)-[^11^C]PK11195, the *V*_T_ determined using 2TCMR and the *V*_T_ assessed by Logan graphical analysis (Fig. 7) showed an excellent correlation (*p* < 0.001, *r* ^2^ = 0.99). Comparison of *V*_T_ values between groups was not possible due to the high interindividual variance (Fig. 8). For example, *V*_T_ values for [^11^C]CB184 in the control rats ranged from 4.42 to 10.47 in the medulla. Therefore, it was decided to use BP_ND_ for the analysis, calculated as the *k*_*3*_/*k*_*4*_ obtained from the 2TCMR (Fig. 5). In control rats, no significant difference was found between the BP_ND_ of [^11^C]CB184 and the BP_ND_ of (*R*)-[^11^C]PK11195. There were statistically significant differences in [^11^C]CB184 BP_ND_ between control and HSE rats for the amygdala (2.4 ± 1.2 vs. 3.6 ± 0.3, *p* = 0.05), hypothalamus (2.7 ± 1.6 vs. 4.6 ± 0.4, *p* = 0.02), medulla (2.6 ± 1.2 vs. 5.1 ± 1.4, *p* = 0.01), pons (2.6 ± 1.3 vs. 4.5 ± 0.9, *p* = 0.02) and septum (1.5 ± 0.9 vs. 2.7 ± 0.5, *p* = 0.02). There was a statistically significant difference in (*R*)-[^11^C]PK11195 BP_ND_ between control and HSE rats only for medulla (1.7 ± 0.6 vs. 2.6 ± 0.4, *p* = 0.02). [^11^C]CB184 BP_ND_ and (*R*)-[^11^C]PK11195 BP_ND_ for the various brain regions in control and HSE rats are shown in Table 5.

**Table 5:** [^11^C]CB184 and (*R*)-[^11^C]PK11195 binding potentials (mean ± SD) in control rats (PBS group) and rats infected with HSV-1 (HSE group) calculated using the reversible two-tissue compartment model

Brain region	[^11^C]CB184		(*R*)-[^11^C]PK11195	
	PBS group	HSE group	PBS group	HSE group
Amygdala	2.39 ± 1.23	3.56 ± 0.33*	1.65 ± 0.62	1.92 ± 0.73
Olfactory bulb	5.56 ± 2.67	8.82 ± 2.97	3.76 ± 1.16	4.03 ± 1.20
Cerebellum	2.88 ± 1.39	3.83 ± 0.63	1.51 ± 0.60	1.89 ± 0.39
Frontal cortex	2.14 ± 1.23	3.08 ± 1.20	1.35 ± 0.51	1.54 ± 0.54
Rest cortices	2.27 ± 1.37	2.69 ± 0.47	1.33 ± 0.46	1.62 ± 0.60
Hippocampus	1.55 ± 0.90	2.44 ± 0.34	0.98 ± 0.44	1.39 ± 0.58
Hypothalamus	2.74 ± 1.62	4.65 ± 0.44*	1.92 ± 0.82	2.28 ± 0.84
Medulla	2.64 ± 1.16	5.10 ± 1.42*	1.65 ± 0.60	2.56 ± 0.42*
Midbrain	1.73 ± 0.98	2.50 ± 0.51	0.96 ± 0.49	1.34 ± 0.42
Pons	2.63 ± 1.31	4.47 ± 0.94*	1.74 ± 0.69	2.44 ± 0.41
Septum	1.53 ± 0.90	2.71 ± 0.46*	1.20 ± 0.38	1.34 ± 0.57
Striatum	1.28 ± 0.74	1.91 ± 0.41	0.88 ± 0.33	1.03 ± 0.43
Thalamus	1.17 ± 0.78	1.85 ± 0.41	0.75 ± 0.35	1.03 ± 0.51
Whole brain	2.25 ± 1.17	3.25 ± 0.41	1.38 ± 0.49	1.72 ± 0.49

*p < 0.05, HSE group vs. PBS group for [^11^C]CB184. There were no statistically significant differences between the PBS groups for [^11^C]CB184 and [^11^C]PK11195

## Discussion

In order to test the suitability of [^11^C]CB184 as a PET tracer for TSPO imaging, we compared [^11^C]CB184 with the most widely used tracer (*R*)-[^11^C]PK11195 in a rat model of HSE. This animal model does not rely on invasive injection of a toxin into the brain, but has known microglial activation in response to viral infection, as confirmed previously by immunohistochemical staining [18, 30]. It can be considered a limitation of the model that the infection cannot be controlled precisely between rats and that the mortality may be relatively high, especially when the rats are followed for longer periods. However, in our study there were no observable differences in clinical symptoms between rats, being limited to slight ruffled fur and/or irritated mouth and nose with the absence of more severe symptoms and premature death. While the levels of neuroinflammation may vary between HSE rats reflecting different levels of HSE severity, [^11^C]CB184 consistently detected a higher TSPO expression in those regions known to be affected in this model, such as the medulla and pons [18].

In the ex vivo biodistribution study of control rats, the highest uptake of [^11^C]CB184 was found in the adrenal glands, heart, kidney, lungs and spleen, when compared with other peripheral regions, while for the brain the olfactory bulb showed the highest uptake. These results are in accordance with those previously reported in mice [23], and with known TSPO expression in these organs [31]. Pretreatment with unlabelled PK11195 resulted in a significant reduction in [^11^C]CB184 uptake in all brain regions in the control rats, except in the cingulate and frontal cortices. In the peripheral organs, pretreatment with unlabelled PK11195 resulted in a statistical significant decrease in all the tissues except fat, plasma and red blood cells. There was a less significant blocking effect in the adrenal glands in the control rats and there was no significant effect in the HSE rats. This apparent low blocking effect of the unlabelled PK11195 in a known TSPO-expressing organ may have been a result of the high variance in the present study (SUV 7.93 ± 5.87, range 4.65 – 19.81, for the control and HSE groups together). Another possible explanation, which is suggested by similar results found with [^11^C]DPA-713 and [^18^F]DPA-714 [18, 32], is the presence of alternative binding sites predominantly expressed in the adrenal glands that do not bind PK11195 [18]. In addition, in the liver there seemed to be a trend towards a higher [^11^C]CB184 uptake in PK11195-pretreated animals, although this difference was not statistically significant. Probably, this observation was a result of decreased liver metabolism due to competition of the tracer with unlabelled PK11195.

The amount of DMSO used as solvent for administration of the unlabelled PK11195 may be considered as a possible confounder in the blocking study. However, DMSO has been shown to have neuroprotective effects, probably mediated via a separate signalling pathway not involving TSPO, and to increase neuronal survival independently of alterations to microglia or astrocytes [33]. Therefore, we consider that the possible interference of DMSO in the blocking effect in the TSPO receptors by the administration of unlabelled PK11195 can be considered minimal or negligible.

The study of the response of microglia to HSV-1 infection, in particular the ability of [^11^C]CB184 to detect changes in TSPO expression, was performed using four different methods: ex vivo biodistribution, analysis of PET images using predefined VOIs, voxel-based analysis of PET images, and comparison of the BP_ND_ calculated from the dynamic PET data using a 2TCMR with a plasma input function. In the ex vivo biodistribution study, HSE rats showed significantly higher SUVs in the amygdala, olfactory bulb, medulla, pons and striatum than control rats. The VOI-based analysis also demonstrated significant differences in HSE rats compared with control rats in most of these regions, including the medulla, pons and striatum, while differences in the olfactory bulb and amygdala were not detected probably due to partial volume effects and/or spillover in these regions. In vivo PET imaging and ex vivo biodistribution data were highly correlated.

In the ex vivo biodistribution study and VOI analysis, possible alterations in TSPO expression were explored bilaterally, making no distinction between brain hemispheres. To explore the existence of asymmetry in [^11^C]CB184 uptake as a consequence of the infection and to explore alterations not limited to predefined anatomical regions, a voxel-based analysis was performed with the same SUV images employed in the VOI analysis. In this voxel-based analysis, a statistically significant difference was found bilaterally in the pons and medulla, and also in the right thalamus and hypothalamus, as well as in regions of the internal capsule, substantia innominata, globus pallidus, diagonal band and bed nucleus of the stria terminalis.

All analysis methods clearly showed an increased expression of TSPO bilaterally in the brainstem (medulla and pons) caused by HSV-1 invasion via the neural pathway from the olfactory bulb to the locus coeruleus, or by direct invasion via the trigeminal nerve [30, 34]. However, the results in other brain regions varied depending on the methodology used. Several factors may be involved in these differences. One factor is that the SUV is a semiquantitative measurement that can be affected by several biological factors (e.g. body size, weight composition, tracer metabolism and blood flow), or technological factors related with the acquisition and reconstruction of the image (e.g. field of view and matrix size) [35]. Moreover, the voxel-based approach may, in theory, identify subtle changes better than VOI-based analysis, as the latter analysis is limited mainly by the spatial resolution of the scanner rather than by the size of the VOIs. In our study, this was reflected in those regions where microglial activation seemed to have a lateralized pattern, as observed in the voxel-based analysis but not in the VOI-based analysis. Moreover, the use of a voxel-based analysis allows investigation of the whole brain and is not limited to hypothesis-based predefined regions. In this study, this advantage led to the higher uptake of [^11^C]CB184 in HSE rats than in control rats in the right globus pallidus, internal capsule and the bed nucleus of the stria terminals, areas that were not included in the VOI-based analysis. Therefore, whenever the study design allows this, it is advisable to perform a voxel-based analysis of parametric images (i.e. voxel-by-voxel representation of the binding, for example using *V*_T_ or BP_ND_, or otherwise SUVs) instead of – or in addition to – a VOI-based analysis.

Both tracers showed similar metabolic rates in plasma, with approximately 50 % of the parent tracer still present in plasma at about 21 min after tracer injection. Interestingly, a significant difference was found at the peak concentration of tracer during the distribution phase at 45 s after tracer injection. While this may have been a consequence of differences in first-pass extraction, binding affinities to plasma proteins [36], differences in lipophilicity, or other intrinsic characteristics of the tracers, this could not be confirmed in the present study. Furthermore, the presence of metabolites of a radioligand in plasma that can cross the BBB may confound the results of PET studies. (*R*)-[^11^C]PK11195 has two major radioactive metabolites, both more polar than the parent (*R*)-[^11^C]PK11195: [^11^C]formaldehyde and *N*-[^11^C]methyl-*sec*-butylamine [37]. The percentage of unchanged (*R*)-[^11^C]PK11195 in rat brain homogenate was 93 ± 4 % and 90 ± 7 % at 20 and 40 min, respectively, after injection [38]. For [^11^C]CB184, on the other hand, the percentage of intact tracer present in the brain at 30 min after injection was approximately 99 %, as confirmed previously in mouse brain [23]. Since the small fraction of [^11^C]CB184 metabolites in brain homogenates probably originated from the blood compartment in the brain, these results suggest that the metabolites of [^11^C]CB184 do not cross the BBB.

The pharmacokinetic analysis of tracer uptake in the brain was performed with a 2TCMR, with plasma corrected for metabolites as the input function. The values of *K*_1_/*k*_2_ were fixed to values for the whole cortex, as it was found to be optimal for the analysis of (*R*)-[^11^C]PK11195 [28]. In this experiment, BP_ND_ seemed to be more appropriate for estimating [^11^C]CB184 and (*R*)-[^11^C]PK11195 binding than *V*_T_, due to interindividual variations of *K*_1_/*k*_2_. Interestingly, no significant significant difference in BP_ND_ between the two tracers was found in the control rats, which seems to indicate that nonspecific binding of the tracers under physiological conditions is similar. In the healthy brain, this diffuse low-level signal is probably attributable to the expression of TSPO in the muscle cells of arteries, perivascular macrophages, lymphocytes and neutrophils, choroid plexus, and ependyma and meninges [39, 40]. HSV-1 encephalitis is known to involve the activation of microglia [41] and astrocytes [42], both of which overexpress TSPO when activated [43]. [^11^C]CB184 was able to detect TSPO overexpression better than (*R*)-[^11^C]PK11195, as reflected by a higher BP_ND_ in the amygdala, hypothalamus, medulla, pons and septum, whereas increased (*R*)-[^11^C]PK11195 uptake was only found in the medulla.

In recent years new radiotracers have been developed for imaging TSPO with PET. The preferred characteristics of these radioligands include [13, 44]:

1. Metabolic stability.
2. High affinity to the target and low nonspecific binding (i.e. good signal-to-noise ratio).
3. Adequate dissociation from the target.
4. Suitable lipophilicity to cross the BBB.
5. Radiolabelled metabolites should not cross the BBB.
6. The synthesis of the radioligand must be simple, quick, and with high yield.

In the present study, the [^11^C]CB184 radioligand was shown to fulfil all these criteria. Its metabolism was similar to that observed for (*R*)-[^11^C]PK11195. Most importantly, the presence of radiolabelled metabolites in brain tissue can be considered negligible. In addition, [^11^C]CB184 showed better specific binding to TSPO than (*R*)-[^11^C]PK11195, e.g. in the medulla, the most affected region in the HSE rats used in this study, the BP_ND_ of [^11^C]CB184 was 93 % higher than in control rats, while the increase in the BP_ND_ of (*R*)-[^11^C]PK11195 was only of 55 %. This was probably the result of the higher affinity of [^11^C]CB184 (7.9 times) for TSPO than (*R*)-[^11^C]PK11195. The pharmacokinetic profile of [^11^C]CB184 also seems to be better than that of (*R*)-[^11^C]PK11195, with a high peak availability of the tracer in the blood pool in the first minute after injection. And finally, the time required for synthesis of [^11^C]CB184 is of about 35 min from the end of irradiation, with a decay-corrected radiochemical yield of 42 ± 7 % (versus 33 ± 15 % for (*R*)-[^11^C]PK11195 [18]).

A significant number of TSPO radioligands have been developed with higher affinity and/or lower nonspecific binding than (*R*)-[^11^C]PK11195, including [^11^C]DAA1106, [^11^C]PBR28 and [^18^F]DPA-714. Our group has previously used the HSE model for evaluation of some of these new TSPO radioligands. [^11^C]DAA1106 did not show significantly higher uptake in vivo in HSE rats than in control rats [45]. Additionally, [^11^C]DPA-713 and [^18^F]DPA-714 were tested in a similar study [18] in which [^11^C]DPA-713 was found to perform better than (*R*)-[^11^C]PK11195, with a similar uptake in infected regions, but with lower nonspecific binding, while [^18^F]DPA-714 uptake was lower than that of (*R*)-[^11^C]PK11195 in infected regions. This last result differs from those obtained in another model of neuroinflammation caused by cerebral ischaemia in which [^18^F]DPA-714 showed a higher signal-to-noise ratio than [^11^C]PK11195 [46]. The differences in methodology in the studies of the new compounds makes direct comparison of the results difficult, and further effort must be focused on the performance of this new generation of TSPO radioligands [47]. Moreover, recent studies have shown mixed binding affinity of several new PET tracers to the TSPO in humans, due to presence of a TSPO polymorphism [21, 22]. Therefore, despite the promising results obtained in this study, further clinical imaging studies with [^11^C]CB184 need to be performed to assess the added value of this new TSPO radioligand, and to determine whether [^11^C]CB184 could replace (*R*)-[^11^C]PK11195.

### Conclusion

Ex vivo and in vivo experiments demonstrated that [^11^C]CB184 shows a high and specific uptake in the encephalitic rat brain. The nonspecific binding of the tracer to healthy brain tissue was comparable to that of (*R*)-[^11^C]PK11195, but [^11^C]CB184 showed a significantly higher uptake in those brain regions affected by the HSE. Our results suggest that [^11^C]CB184 could be a good alternative for the imaging of TSPO overexpression in neuroinflammatory processes, and further evaluation in humans is warranted.
